# The Potential Intermediate Hosts for SARS-CoV-2

**DOI:** 10.3389/fmicb.2020.580137

**Published:** 2020-09-30

**Authors:** Jie Zhao, Wei Cui, Bao-ping Tian

**Affiliations:** Department of Critical Care Medicine, The Second Affiliated Hospital, Zhejiang University School of Medicine, Hangzhou, China

**Keywords:** SARS-CoV-2, intermediate host, transmission, COVID-19, review

## Abstract

The coronavirus disease 19 (COVID-19) caused by severe acute respiratory syndrome coronavirus 2 (SARS-CoV-2), has become a global pandemic since the first report in Wuhan. COVID-19 is a zoonotic disease and the natural reservoir of SARS-CoV-2 seems to be bats. However, the intermediate host explaining the transmission and evolvement is still unclear. In addition to the wildlife which has access to contact with bats in the natural ecological environment and then infects humans in wildlife market, domestic animals are also able to establish themselves as the intermediate host after infected by SARS-CoV-2. Although recent studies related to SARS-CoV-2 have made a lot of progress, many critical issues are still unaddressed. Here, we reviewed findings regarding the investigations of the intermediate host, which may inspire future investigators and provide them with plenty of information. The results demonstrate the critical role of the intermediate host in the transmission chain of SARS-CoV-2, and the efficient intervention on this basis may be useful to prevent further deterioration of COVID-19.

## Introduction

**FIGURE 1:**
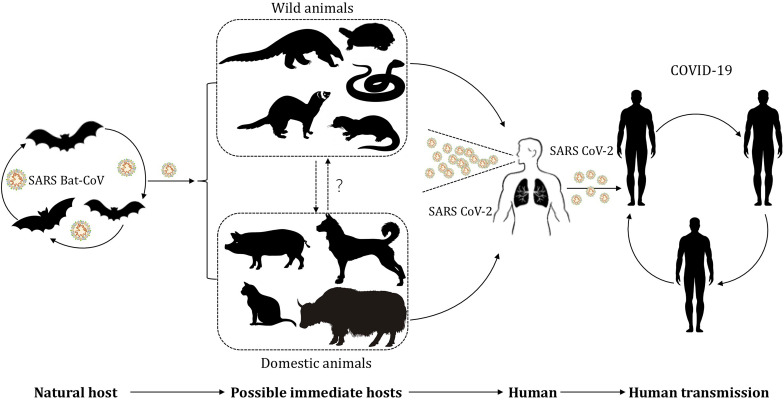
The potential transmission of SARS-CoV-2 between hosts and humans. SARS-CoV-2, originated from bat-nCoV, infected wild animals and gradually evolved in the intermediate host after mutation and recombination. Wildlife business give chance for SARS-CoV-2 to infect humans and domestic animals. SARS-CoV-2 induce a pandemic in human population by respiratory droplet transmission and close contact transmission.

The current global pandemic of coronavirus disease 19 (COVID-19) is attributed to the transmission of a pathogen named SARS-CoV-2 (severe acute respiratory syndrome coronavirus 2). In the early stage of the outbreak of COVID-19, Shi et al. (2020) found, using a sequence homology comparison at the whole-genome level, that SARS-CoV-2 in samples obtained from seven patients shared 96.2% sequence identity with bat-coronavirus (bat-nCoV) RaTG13 (Zhou P. et al., 2020). Besides, Zhou H. et al. (2020) reported another bat-nCoV denoted RmYN02, which shared 93.3% identity with SARS-CoV-2 at the scale of complete genome. Based on biological features of bat and the high identity sequence between bat-nCoV and SARS-CoV-2, bats are considered as the natural reservoir of SARS-CoV-2 for now (Chan et al., 2013; Nguyen et al., 2020; Ren et al., 2020b; World Healthy Organization, 2020; Ye et al., 2020; Zhou H. et al., 2020; Zhou P. et al., 2020). However, the intermediate host, where SARS-CoV-2 acquires some or all of the mutations necessary for efficient transmission in humans, remains uncertain. During the investigation of the transmission route for COVID-19, a connection was observed between the early reported cases and the Huanan seafood market in China, where a variety of wild animals in addition to seafood were available for purchase at the time of the outbreak (Wu F. et al., 2020). Although not all early cases were associated with this market, it is apparent that transactions involving wildlife accelerate the transmission of the zoonotic infectious disease (Zhang and Holmes, 2020). Discovery of the potential intermediate host is beneficial for us to cut the transmission between animals and humans via timely and effective intervention, and thus helps prevent this pandemic further deteriorating and decreases loss of life and property. In order to provide information and motivate brainwave for investigators, we will summarize and analyze up-to-date findings of the potential hosts of SARS-CoV-2 (Figure 1).

## Betacoronavirus Family and Characteristic

Currently, SARS-CoV-2 is a member of the genus Betacoronavirus family, of which the severe acute respiratory syndrome coronavirus (SARS-CoV) and the Middle East respiratory syndrome coronavirus (MERS-CoV) have caused epidemics in humans before (Lu R. et al., 2020; Zhang and Holmes, 2020). The coronavirus is composed of single strand RNA, non-structural proteins (nsp) and structural proteins such as Spike (S) protein, Envelope (E) protein, Membrane (M) glycoprotein, Nucleocapsid (N) protein (Su et al., 2016; Wu J. et al., 2020). Bat-nCoV is viewed as the origin of MERS-CoV and SARS-CoV (de Wit et al., 2016; Cui et al., 2019). Consistently, bats are considered as the natural host of SARS-CoV-2 (Chan et al., 2013; Ren et al., 2020b; Zhou P. et al., 2020). Generally speaking, the intermediate host of SARS-CoV is masked palm civet, whereas the intermediate host of MERS-CoV is dromedary camel (de Wit et al., 2016). Host receptors mediate the entry of SARS-CoV-2 via binding to the receptor-binding domain (RBD) of S protein. MERS-CoV uses dipeptidyl peptidase 4 (DPP4, also known as CD26) whereas SARS-CoV utilizes angiotensin-converting enzyme 2 (ACE2) (Kuhn et al., 2004; Raj et al., 2013). Sequence comparison indicates that SARS-CoV-2 shows 79% identity with SARS-CoV and 50% similarity to MERS-CoV, respectively (Lu R. et al., 2020; Ren et al., 2020a; Zhang and Holmes, 2020). Interestingly, SARS-CoV-2 also recognizes human ACE2 (hACE2), which can be utilized by SARS-CoV. In terms of the crystal structure, the conformation of the RBD-ACE2 complex is more compact in SARS-CoV-2 than that in SARS-CoV (Golden et al., 2020; Hoffmann et al., 2020; Shang et al., 2020). The RBD of SARS-CoV-2 is composed of a core and an external subdomain, the latter is similar to that of SARS-CoV, though several key residues responsible for the binding of RBD and ACE2 receptor are variable (Lu R. et al., 2020). Recently, Lan et al. (2020) suggested that the resolved structure of the S protein bound to hACE2 receptor have 20 key AAs in hACE2 for interacting with receptor-binding motif (RBM). Investigation of the interaction between RBD and ACE2 is a hotspot for now, because of its benefits in exploring the viral mechanisms and searching the potential intermediate host. However, regarding ACE2 as the unique receptor of SARS-CoV-2 is unreasonable for now. It has been reported that basigin (BSG)/CD147 could also mediate SARS-CoV-2 entry. Besides, some investigators refuted the direct interaction between CD147 and RBD, suggesting more studies are needed to clarify the mechanism in detail (Shilts and Wright, 2020; Wang K. et al., 2020). The direction of exploring other potential viral receptors should not be ignored in further studies.

## Wildlife as the Potential Intermediate Hosts

### Pangolin

#### Sequence Similarity Between Coronaviruses From Pangolin and SARS-CoV-2

Since Xiao et al. (2020) found viral strains isolated from the infected Malayan pangolins with clinical signs, histological changes and circulating antibodies shared 90.1% identity with SARS-CoV-2, whether the pangolin participates in the transmission of COVID-19 as an intermediate host has become a hotspot of debate. In the report of Xiao et al. (2020), the E, M, N, and S genes encoding structural proteins between pangolin coronavirus (pangolin-nCoV) and SARS-CoV-2 showed 100, 98.6, 97.8, and 90.7% amino acid identity, respectively. Lam et al. (2020) also detected high similarity (85.5–92.4%) of viral sequence between pangolin-nCoV and SARS-CoV-2, whose samples came from smuggled pangolins intercepted in Guangxi during the same period. So far, independent sequence comparison conducted by Liu P. et al. (2020) and Zhang T. et al. (2020), found a pangolin-nCoV sharing 90.32, 91.02% similarity to SARS-CoV-2, respectively. Besides, the corresponding phylogenetic tree based on Hausdorff distance and Center distance between SARS-CoV-2 strains and host-nCoV groups confirmed the close relation between pangolin-nCoV and SARS-CoV-2 (Dong et al., 2020). With regard to high similarity to SARS-CoV-2, pangolin-nCoV is only secondary to bat-nCoV (Supplementary Table 1). However, several investigators found those published results were might origin from the same pangolin-nCoV source when they analyzed the dataset used by Chan and Zhan (2020). It raises a question of whether pangolin is really intermediate host mediating cross-species transmission or just an incidental host contracting the coronavirus during trafficking.

#### Genetic Evolvement Analysis and CpG Values Comparison

The zinc finger antiviral protein (ZAP), an important mammalian antiviral protein, can bind specifically to CpG dinucleotides and degrade viral genomes (Meagher et al., 2019). Many mammalian RNA viruses avoid immune defense by evolving CpG deficiency in a special cellular environment (Greenbaum et al., 2009; Takata et al., 2017). Thus, the CpG values is a special genomic landmark prompt viral evolution history and host trajectory. MacLean et al. (2020) detected that RmYN02 is recombined between the high and low CpG lineages of bat-nCoV, indicating a co-infection and evolution in bats without the involvement of other species. Besides, CpG levels of SARS-CoV-2 and RmYN02 only differ in the recombinant region. This suggests that the adaptive shift in CpG composition has finished before recombination events, in a word, diversifying selection imprints and ancestral CpG depletion are at the base of bat-nCoV lineage (MacLean et al., 2020). Therefore, events in SARS-CoV-2 evolution occurred in bats before spilling over into humans. Pangolin might be just an opportune host intermediating bat-to-human SARS-CoV-2 jump and entry. Investigators consequently devoted themselves to completing the epidemiological survey of data of pangolin to clarify when and where healthy pangolins get infected. Detection of viral nucleic acid was conducted in Sunda pangolins (*Manis javanica*), which were either confiscated from smugglers or rescued from the wild between August 2009 and March 2019. These pangolins were reported to be destined for Southeast Asian countries to China, and no sample yielded a positive PCR result for targeted coronaviruses (Lee et al., 2020). Contrast to the infected pangolins in China, these pangolins drawn from an “upstream” cohort of animals yet to enter or just entered the illegal trade network have not been attacked by coronavirus. Those pangolins were healthy when captured in wild and got infected when contacting with other wildlife during transportation and transaction. So far, we still cannot deny the possibility of pangolin origin since the number of samples we have analyzed is too limited compared to the diversity of pangolin-nCoV in nature.

#### Key Residues Analysis on the Interface of Interaction Between RBD and ACE2 Receptor

Although Bat-nCoV RaTG13 share 96.2% similarity with SARS-CoV-2, the identity of RBD to SARS-CoV-2 is inferior to pangolin-nCoV. The analysis of pangolin-nCoV metagenomic dataset and the multiple alignments across the RBM segments revealed that pangolin-nCoV shared 89% nucleotide similarity and 98% amino acid identity to SARS-CoV-2 (Wong et al., 2020). The difference in key amino acids of RBD between Malayan pangolin-nCoV and SARS-CoV-2 is just one amino acid. Regarding bat-nCoV RaTG13, four positions of five key amino acids are different in the RBD region (Xiao et al., 2020; Zhou P. et al., 2020). One explanation is that bat-nCoV was transmitted from bat to pangolin by chance, and got recombined with pangolin-nCoV in pangolin, after which the new recombined virus acquired the ability to invade human cells. Lopes et al. (2020) regarded the ACE2 as the barrier for cross-species transmission and conducted a host evolutionary analysis of ACE2 sequence. They found the evolutionary divergence between pangolin ACE2 and hACE2 is relatively lower than that between bat ACE2 and hACE2 (Lopes et al., 2020). Interestingly, according to a category ranking score based on the conservation properties of 25 amino acids important for the binding, pangolin and bat, respectively, scored low and very low (Damas et al., 2020). The contradiction in the above two studies might be attributed to the different analysis method. Thus, the accuracy of host prediction method needs further biological experiments to confirm. The ACE2 is not the only receptor that mediating SARS-CoV-2 entry. The conclusion based on such analysis design deserves further debate (Wang K. et al., 2020).

#### Cellular Experiments Mimic the Interaction Between ACE2 and S Protein

Tang et al. (2020) conducted virus infectivity studies using HEK293T cells expressing ACE2 from 11 species of animals, and found that pangolin-ACE2 could mediate SARS-CoV-2 entry. Besides, ACE2 of *Rhinolophus ferrumequinum* cannot support the entry of SARS-CoV-2 that is a capability of ACE2 of *Rhinolophus sinicus* in experiments conducted by Tang et al. (2020) and Zhou P. et al. (2020). By using pseudotyping particles of SARS-CoV-2 Spike mimics particle entry and quantitative cell-cell fusion assay, Conceicao et al. (2020) observed that pangolin-ACE2 sustained higher levels of entry than hACE2. Additionally, they also found dramatic difference in the ability of SARS-CoV-2 Spike to utilize ACE2 among different bat types (Conceicao et al., 2020). The above results supported the high susceptibility of pangolin, but also indicated that diverse bat types could present different sensitivity. Nevertheless, several limitations can be found *in vitro* transfection experiments. Firstly, artificial expression level of ACE2 has not been able to match the physiological status. In fact, example as hACE2, ACE2 expressed the level of the same individual is divergent in different organs and cell types (Chen J. et al., 2020; Wang Y. et al., 2020). Thus, it is unilateral to interpret the result just depending on the *in vitro* transfection experiments. Besides, the possibility that SARS-CoV-2 utilizes other cellular receptor except ACE2 to invade bats and/or pangolins still cannot be excluded. We should notice that viral evolvement is a complicate process and there are possibilities of SARS-CoV-2 mediating different receptors into different species.

#### The S1/S2 Cleavage Site in S Protein

The S protein played a crucial role in determining host tropism and transmission capacity. It consists of S1 domain mediating receptor binding and S2 domain responsible for cell membrane fusion. With the help of cellular protease such as transmembrane protease serine2 (TMPRSS2), efficient proteolytic processing of S protein at the S1/S2 cleavage site is also important in mediating SARS-CoV-2 entry, after which S2 subunit will allow fusion of viral and cellular membranes (Hoffmann et al., 2020). The characteristic S1/S2 cleavage site might be a critical factor impacting host range and transmissibility. Li X. et al. (2020) re-analyzed the human SARS-CoV-2 and found a unique peptide (PRRA) insertion region in the S protein at the junction of S1 and S2 junction inducing a furin cleavage motif (RRAR). Comparing to wild type counterparts, the deletion of PRRA from SARS-CoV-2 indeed reduced fusogenic ability in the presence of ACE2 (Liu S. F. et al., 2020). However, the PRRA insertion was not found in the two samples obtained from previously reported Malayan pangolin-nCoV sequencing data, opposing the view that pangolin is a direct intermediate host (Li X. et al., 2020).

### Mink

Guo et al. (2020) proposed a virus host prediction (VHP) method based on a deep learning algorithm, and found that bat and mink viruses had the closest infectious patterns to SARS-CoV-2. It not only provides us with a new method to investigate the potential intermediate host but also arouses our attention to mink. Mink has been domesticated for a long history, and now is farmed for their fur in some regions. Recently, SARS-CoV-2 has attacked several mink farms in Netherlands, in which more minks were dying than usual, and some had respiratory symptoms. An epidemiological investigation in two mink farms showed that in both farms, the virus was introduced by a farm worker who had COVID-19, which indicated the transmission from human-to-mink (Oreshkova et al., 2020). Besides, genetic and epidemiological sleuthing also showed that at least two farm workers had caught the virus from mink, and stray cats in the surroundings of the farms have been infected as well, indicating the existence of animal-to-human and animal-to-animal transmission (Enserink, 2020). Airborne transmission occurs in two methods. A sneeze or a cough can cause droplet sprays, of which the diameter is larger than 5 μm. Another method is spreading through aerosol (smaller than 5 μm in diameter), in which SARS-CoV-2 can maintain viable and infectious for hours (Asadi et al., 2020; van Doremalen et al., 2020). Thus, detection of viral RNA in airborne inhalable dust in the mink farm alerted that farm workers should wear protective equipment to avoid being attacked by dust and/or droplets (Enserink, 2020). This event not only depicted the transmission chain as human-to-mink, mink-to-mink, and mink-to-human/or other animals, but also demonstrated the indirect transmission method as dust or droplet, which is the potent evidence to support that mink can serve as the intermediate host for SARS-CoV-2.

### Turtle

Liu Z. et al. (2020) suggested that turtle such as *Chrysemys picta bellii*, *Chelonia mydas*, and *Pelodiscus sinensis* are potential intermediate hosts based on systematic comparisons and the divergence of interaction between the RBD and the ACE2 receptor. However, Zhang C. et al. (2020) refuted the above opinion as they analyzed the affinity to S protein of the 20 key residues in ACE2 and discovered nearly half of the key residues in ACE2 from turtle were abolished (Luan J. et al., 2020a). Because no coronavirus isolated from turtle is used to gene sequence comparison, the recent investigations majored in evaluation of interaction between RBD and ACE2. However, the position of the critical residues in the interaction is still controversial, not to mention considering the impact of interaction force between residues in dynamic process. It is too early to confirm or deny the intermediate host status of turtle.

### Snake

On such a premise that virus acquires the capability of efficient replication by evolving similar codon usage pattern to its hosts, the high similarity of the relative synonymous codon usage (RSCU) bias between SARS-CoV-2 and snakes found by Ji et al. (2020), suggested that snake might be an intermediate host, in which SARS-CoV-2 evolved and get the ability to infect humans by homologous recombination. However, the reliability of the result is under doubt, as the experiment itself has several shortages such as limited protein coding sequences, limited vertebrate species and outdated codon usage database. Besides, algorithm analysis re-implemented by Zhang C. et al. (2020), indicated frogs, not snakes were the vertebrates sharing the lowest RSCU distance to SARS-CoV-2, suggesting the result of Ji et al., at least incomplete. More importantly, the rationality of RSCU analysis to identify the viral host deserves debate. Many studies have found that the RSCU of virus is not always similar to that of its host. In fact, viral codon usage bias involves mutation pressure, particular DNA/RNA or protein structure and genome size, which is more complicated than parasitic adaptation (Gong et al., 2020). Therefore, it is inappropriate to infer host on the basis of viral condon usage bias attributed only to host adaptation. Another study compared the key residues of snake-ACE2 and hACE2, and found snake-ACE2 lost the capability to interact with the RBD as half of the key residues were abolished (Luan J. et al., 2020a). In summary, no direct experimental evidence determines that snakes can be infected with SARS-CoV-2 and act as the viral host.

### Ferrets

According to current understanding, ferrets are susceptible to infect SARS-CoV, whereas at the same time avoid the MERS-CoV invasion (Martina et al., 2003; Raj et al., 2014). Kim et al. (2020), detected SARS-CoV-2 and pulmonary histopathological changes in ferrets contacting with SARS-CoV-2 directly or indirectly. Another experiment intranasally inoculating SARS-CoV-2 on ferrets which was conducted by Shi et al. (2020) revealed that ferrets have high susceptibilities to SARS-CoV-2. Both animal studies supported that ferret acts as a potential host of SARS-CoV-2. However, experiments of SARS-CoV pseudotype entry and cell-cell assays indicated that ferret ACE2 was not used efficiently by SARS-CoV-2 for entry (just 1% of human levels) even if ferret ACE2 can support SARS-CoV-mediated fusion (Conceicao et al., 2020). The controversy in above studies exposes the drawback when we assess the host susceptibility just relying on *in vitro* transfection experiments. Signal cell entry simulation cannot represent comprehensive reaction involving a complicated immune system, let alone current condition is not close enough to natural physiological state. Can ferret ACE2, for which receptor usage *in vitro* is poor, really assume responsibility of established *in vivo* infection? Is there any possibility that ferret infection of SARS-CoV-2 depends on other receptors? These questions need more associated investigations to answer.

## Domestic Animals as the Potential Intermediate Hosts

### Case Reports and Epidemiological Investigations

Deng et al. performed an antibody survey among 1914 serum samples from 35 species and did not detect any SARS-CoV-2 specific antibody. They consequently draw a conclusion that those species can be excluded from the candidates for the intermediate host (Deng et al., 2020). COVID-19 prevalence in region of sample source is an important reference to judge the reliability of the conclusion. The above results can be explained by low SARS-CoV-2 exposure level, in other words, SARS-CoV-2 had not been widely circulating in a local animal community. It was reported that two dogs and a cat from a family with confirmed COVID-19 human cases got SARS-CoV-2 infection (Chini, 2020). Besides, tigers and lions were also detected as SARS-CoV-2 positive, which was associated with positive zoo keepers (McAloose et al., 2020; Wang L. et al., 2020). These facts alert us that human-to-animal transmission of SARS-CoV-2 may exist. However, we cannot exclude the possibility that the dog did not get infected from its owner but from other viral sources, which might also infect its owner. In addition, whether infected dogs can transmit the virus back to humans or other animals remains unclear (Mallapaty, 2020). Another survey performed by Sarah T, which tested 21 domestic pets (9 cats and 12 dogs) living in community with positive patients, indicated that viral sequence and serologic antibody of all pets are both negative (Temmam et al., 2020). They evaluated the pets’ susceptibility to SARS-CoV-2 under exposure conditions of a natural environment, and suggested a low transmission rate between humans and pets, below a reproduction number of 1. The drawback of this research is apparent, not only the sample size and animal type are insufficient, but also the environmental viral load is uncertain. More surveys should be carried out as the situations vary from community to community. Notably, the infected animals remained asymptomatic during quarantine, indicating that post-infection animals might present different symptoms and virus transmission patterns comparing to humans (Sit et al., 2020). This may increase the difficulty to recognize the infected animals accurately, and alert the family with confirmed patients to keep an appropriate social distance with their pets.

### Evaluate Affinity of ACE2 From Different Species to Bind S Protein Based on key Residues Comparison

Based on the view that key atomic-level interaction for coronavirus invasion is between the RBD and host ACE2 receptor, lots of studies were performed surrounding key residues (Wan et al., 2020). Most of the key residues used by ACE2 for recognizing the S protein of SARS-CoV-2 have been found in many domestic mammals, suggesting a high susceptibility of SARS-CoV-2 (Luan J. et al., 2020b). Phylogenetic clustering and sequence alignment performed by Qiu et al. (2020), whose aim was to evaluate the invasive capability of SARS-CoV-2 to ACE2 in various species, also revealed that domestic animals such as cat, cow, buffalo, goat, sheep, pigeon, and swine ACE2s might be utilized by SARS-CoV-2. Dabravolski and Kavalionak detected that spike glycoprotein of yak-delivered betacoronavirus showed the highest similarity to the SARS-CoV-2 (Dabravolski and Kavalionak, 2020). Additionally, the analysis of the affinity to S protein of the 20 key residues in ACE2 conducted by Zhang C. et al. (2020), illustrated that ACE2 from Bovidae maintain the capability to interact with RBD as the majority of key residues in ACE2 identical to hACE2 (Luan J. et al., 2020a). These results remind us that yak, belonging to Bovidae, may be an intermediate host of SARS-CoV-2. So far, most researches major in structure similarity between host ACE2 and hACE2. Actually, the binding capability between S protein and ACE2 is complex and influenced by many factors. The ACE2 with low structural similarity to hACE2 may have high functional similarity to hACE2 if certain critical residue mutation enhances the binding capability. Besides, intraspecies variation might also be a crucial component of reservoir competency that we cannot assess. Frank et al. (2020) analyzed ACE2 sequences from 198 species (90 bat species) and found that mammals, particularly bats are diverse at residues contacting SARS-CoV-2. These findings demonstrate that all individuals in the same species might present different susceptibility, which makes host identification more difficult. Besides, the individual has an intact immune system preventing the attack of the virus. Therefore, there is still a long way to investigate further. Zhai et al. (2020) observed the X-ray structures of hACE2 bound to the RBD from SARS-CoV-2 and tried to define the crucial residues for binding by comparing the interaction sites of ACE2 proteins of various species. They found the binding of RBD to ACE2 tolerated a surprisingly large number of amino acid changes in the interaction surface for species known to support SARS-CoV-2 infection, suggesting a relatively low species barrier for viral transmission (Zhai et al., 2020). These animals, especially those close to humans, are at a high risk of becoming an additional animal reservoir once they contracted SARS-CoV-2 after infection.

### Assess Interaction Between SARS-CoV-2 and ACE2 From Various Species

Functional assays of ACE2 protein using authentic SARS-CoV-2 conducted by Liu Y. H. et al. (2020), suggested 44 mammalian ACE2 orthologs including domestic animals could support viral entry. They found that the ACE2 protein of New World monkeys cannot bind S protein and mediate viral entry. It is consistent with the results of inoculated experiments that New World monkeys were resistant to SARS-CoV-2 infection (Lu S.Y. et al., 2020). Liu Y. H. et al. (2020) mutated several critical residues and found that Hela cell transduced by humanized ACE2 orthologs from monkeys is permissiveness to infection. It sheds light on the crucial residues in ACE2 protein determining the host susceptibility, which will help establish an experimental animal model and identify the potential intermediate host. Li Y. J. et al. (2020) used expressed mouse IgG2 Fc fusion RBD proteins to perform surface staining of cells transfected with expression plasmids for the seventeen ACE2 orthologs, and found domestic animals such as camels, cattle, horses, goats, sheep, pigs, cats, and rabbits supported the efficient entry of SARS-CoV-2, SARS-CoV, and Bat-nCoV RaTG13. Screening a host cannot just rely on evaluating the viral capability of entry. More importantly, whether SARS-CoV-2 can stay and replicate in such an animal and eventually spillover to another specie. For example, the intermediate host of SARS-CoV is civet, although it might have a relatively broad potential hosts range, according to de Wit et al. (2016) and Li Y. J. et al. (2020). Absorbingly, pigs and dogs appeared less susceptible to SARS-CoV-2 in the intranasally inoculated experiment even if their ACE2 proteins can function as SARS-CoV-2 receptor (Shi et al., 2020). Zhai et al. (2020) investigated the level of ACE2 expression in different organs and considered that the low susceptibility of pigs and dogs was attributed to the relatively low mRNA levels in the respiratory tract. In terms of hACE2, it is mainly expressed in the small intestine, kidney etc., rather than lung, let alone mRNA levels cannot reflect the abundance of ACE2 protein (Chen J. et al., 2020; Wang Y. et al., 2020). Although lacking high expression of ACE2 in the lung, humans also have a high susceptibility to SARS-CoV-2. This fact suggests that elevation of ACE2 expression does not mean a higher susceptibility to SARS-CoV-2. Among the investigated animals, cats have the highest proportion of ACE2 and TMPRSS2 co-expressed cell regarded as SARS-CoV-2 target cell by Shi et al. (2020). In addition to the high susceptibility of cats, they also highlighted multiple infected routes because of the wide distribution of ACE2 and TMPRSS2 co-expressed cells among digestive system and urinatory system (Chen D. S. et al., 2020). However, the above inferences have a noticeable shortage, as the ACE2 expression and distribution cannot be linked to the capability of viral invasion simply. ACE2 is a key counterregulatory enzyme deattenuating the effects of angiotensin II activity part responsible for organ injury. Thus, SARS-CoV-2 will down-regulate ACE2 expression to avoid its protective effects (Vaduganathan et al., 2020). Interpretation of ACE2 expression levels is complex, which need further investigation.

### SARS-CoV-2 Inoculated Experiment and Animal Contact Test

Shi et al. (2020) intranasally inoculated SARS-CoV-2 in several domestic species and found cats are highly susceptible, but dogs, pigs, chickens, and ducks had less susceptibility. Consistent with Shi et al. (2020), cats those intranasally inoculated SARS-CoV-2 or close contacted with infected cats were detected got infected. Additionally, a robust neutralizing antibody response occurs rapidly and help prevent a second infection of SARS-CoV-2. Notably, infected cats will shed virus for no more than 5 days without clinical symptoms, while humans can shed virus for more than 3 weeks. So, if cats staying with its symptomatic owner followed appropriate quarantine procedures, there is a low possibility for cats to infect another human (Bosco-Lauth et al., 2020). On the one hand, direct intranasal inoculation of the high dose of virus used in Shi et al. (2020) experiment rarely happened within the normal social distance between owners and pets. However, overly intimate behaviors between owners and pets are common in contemporary society, which substantially increased exposure risks. Besides, stray dogs and cats always aggregate and contact with each other to compete for territory or food. Since pets are always important members of our family, more studies need to be carried out to clarify whether the pets living with us can spread the virus or not. Besides, stray cats and dogs also deserve attention, because they always wander around humans, especially in remote villages, where the public health system is relatively weaker.

## Conclusion

Currently, the bat is the natural host for SARS-CoV-2 according to mainstream perspectives, but the intermediate host of SARS-CoV-2 is still unclear (Supplementary Table 1). The intermediate host is not just the bridge that links natural origin and susceptible population, but also the viral factory where SARS-CoV-2 evolve gradually and replicate massively. Recognition of the intermediate host is of much significance in cutting off the transmission chain and preventing the COVID-19 pandemic from deteriorating. So far, strains of SARS-CoV-2 have been shown to have relatively high similarity to pangolin-nCoV, only secondary to bat-nCoV. Thus, the pangolin is a highly suspected candidate of the intermediate host. Other wild animals such as snakes, minks and turtles should not be ignored because they are also found in wildlife markets and present a high risk for infection. Due to Chinese eating habits and historical tradition, wild snakes and turtles are used as food and medicine in some regions, alerting scientists to find relevant evidence to support or oppose the possibility (Table 1). The high susceptibility of ferrets to SARS-CoV-2 also deserves our attention. Additionally, domestic animals, especially companion animals should not be excluded yet, because it is not only a scientific problem but also a social problem once certain companion animal are confirmed as the intermediate host. It may cause panic in population and induce behavior of discarding. The government will face new challenges in social management and maintaining appropriate social distances with suspected animals. In a word, many critical issues in exploring the intermediate host of SARS-CoV-2 are still uncertain. Further studies investigating sufficient samples will provide more information about the intermediate host, the way of blocking transmission, and yielding effective prevention. Determining animal hosts is an important stage in the chain of transmission of a zoonotic disease. It is especially crucial to protect wildlife and standardize wildlife management, through which we can build a sound ecological environment and prevent pandemic disease deteriorate.

**TABLE 1:** The potential intermediate hosts for SARS-CoV-2.

**Species**	**Analytic method**	**Supported evidence or opposed reason**	**References**
Pangolin	Gene Sequence Analysis and Comparison	Malayan pangolins contain sequences strongly similar to SARS-CoV-2	Xiao et al., 2020
	High-throughput Sequencing and phylogenetic analysis	Pangolin-nCoV belongs to two sub-lineages of SARS-CoV-2 related coronaviruses	Lam et al., 2020
	Molecular and phylogenetic analyze the assembled complete genome of pangolin-nCoV	Pangolin-nCoV have the highly conserved S genes and structure of RBD protein to SARS-CoV-2	Liu P. et al., 2020
	Gene sequence analysis of coronavirus genomes reconstructed from viral metagenomic datasets of hosts and SARS-CoV-2	High sequence similarity in the RBM between SARS-CoV-2 and a coronavirus genome from datasets of pangolin	Wong et al., 2020
	Systematic comparison and analysis to predict the interaction between the RBD and the ACE2	Regarding the similarity of the key amino acids of interaction between RBD and ACE2 to humans, the pangolin is closer than the bat	Liu Z. et al., 2020
	Molecular evolution and phylogenetic analysis of SARS-CoV-2 and hosts ACE2 protein	The evolutionary divergence between pangolin ACE2 and hACE2 is lower than that between bat ACE2 and hACE2	Lopes et al., 2020
	The phylogenetic tree based on Hausdorff distance and Center distance between SARS-CoV-2 strains and host-nCoV groups	The pangolin-nCoV is closely related to the SARS-CoV-2 group based on the genome divergences	Dong et al., 2020
	Phylogenetic, split network, transmission network, and comparative analyses of the genomes	The pangolin-nCoV from the two pangolin samples did not have the PRRA insertion, which is crucial in viral invasion	Li X. et al., 2020
	Virus infectivity studies using HEK293T cells expressing ACE2 from 11 species of animals	Pangolin ACE2 could mediate SARS-CoV-2 entry	Tang et al., 2020
	Pseudotyping particles of Spike mimics particle entry and quantitative cell-cell fusion assay	Pangolin sustained higher levels of entry than was seen with an equivalent hACE2 construct	Conceicao et al., 2020
Mink	Compare the infectivity patterns by deep learning algorithm of VHP	Mink coronavirus have the closest infectious patterns to SARS-CoV-2	Guo et al., 2020
	Genetic and epidemiological sleuthing	The SARS-CoV-2 outbreak in mink farms is introduced by humans, and infected minks can transmit the virus to human and other animals via viral dust or droplets	Oreshkova et al., 2020
Turtle	Systematic comparison and analysis to predict the interaction between the RBD and ACE2	Regarding the similarity of the key amino acids of interaction between RBD and ACE2 to humans, the turtle is closer than the bat	Liu Z. et al., 2020
	Analyze the affinity to S protein of the 20 key residues in ACE2	**Oppose:** Nearly half of the 20 key residues in ACE2 from turtles were abolished	Luan J. et al., 2020a
Snake	Relative synonymous codon usage (RSCU) comparison and analysis	Snake shared the lowest RSCU distance to SARS-CoV-2	Ji et al., 2020
	Analyze the affinity to S protein of the 20 key residues in ACE2	**Oppose:** Nearly half of the 20 key residues in ACE2 from snakes were abolished	Luan J. et al., 2020a
	Reperform the RSCU comparison and analysis conducted by Ji et al. (2020)	**Oppose:** RSCU is not specific enough to identify the intermediate host	Zhang C. et al., 2020
Ferrets	Establish a ferret model of SARS-CoV-2 infection and transmission	SARS-CoV-2 is effectively transmitted to naïve ferrets by direct contact and leads acute bronchiolitis	Kim et al., 2020
	Intranasally inoculated SARS-CoV-2 to domestic animals	Ferrets have high susceptibility to SARS-CoV-2	Shi et al., 2020
	Pseudotyping particles of Spike mimics particle entry and quantitative cell-cell fusion assay	Ferret ACE2 is not used efficiently by SARS-CoV-2 for entry	Conceicao et al., 2020
Bovidae (yak)	Analyze the affinity to S protein of the 20 key residues in ACE2	The majority of key residues in ACE2 are identical to hACE2 protein	Luan J. et al., 2020a
	Phylogenetic tree analysis and structural models’ comparison	A yak betacoronavirus strain has spike glycoproteins structure models closest to SARS-CoV-2	Dabravolski and Kavalionak, 2020
Dogs	Pseudotyping particles of Spike mimics particle entry and quantitative cell-cell fusion assay	Dog sustained higher levels of entry than was seen with an equivalent hACE2 construct	Conceicao et al., 2020
	Intranasally inoculated SARS-CoV-2 to domestic animals	**Oppose:** Dogs have little susceptibility to SARS-CoV-2	Shi et al., 2020
	Investigate the level of ACE2 expression in different organs	**Oppose:** Higher mRNA levels in organs such as kidney and heart, while low mRNA levels in respiratory tract	Zhai et al., 2020
	Use single-cell technique to screen of ACE2 and TMPRSS2(SARS-CoV-2 target cell) in different organs of animals	**Oppose:** Co-expression of ACE2 and TMPRSS2 is absent in poultry lung cells and rare in dog lung cells	Chen D. S. et al., 2020
	Virus infectivity studies using HEK293T cells expressing ACE2 from 11 species of animals	Dog ACE2 could mediate SARS-CoV-2 entry	Tang et al., 2020
Cats	Intranasally inoculated SARS-CoV-2 to domestic animals	Cats have high susceptibility to SARS-CoV-2	Shi et al., 2020
	Phylogenetic clustering and sequence alignment to evaluate the receptor-utilizing capability of ACE2	Cat ACE2 have the receptor-utilizing capability of SARS-CoV-2	Qiu et al., 2020
	Use expressed RBD proteins to perform surface staining of cells transfected with expression plasmids of ACE2 orthologs	Cats support the efficient entry of SARS-CoV-2, SARS-CoV, and Bat-nCoV RaTG13	Li Y. J. et al., 2020
	Intranasally inoculated SARS-CoV-2 or close contact with infected cat	Cats are subclinical infection and shed virus for no more than 5 days	Bosco-Lauth et al., 2020
	Pseudotyping particles of Spike mimics particle entry and quantitative cell–cell fusion assay	Cat sustained higher levels of entry than was seen with an equivalent hACE2 construct	Conceicao et al., 2020
	Use single-cell technique to screen of ACE2 and TMPRSS2(SARS-CoV-2 target cell) in different organs of animals	Cats have the highest proportion of SARS-CoV-2 target cell, and those cells were widely distributed among digestive system, respiratory system and urinatory system	Chen D. S. et al., 2020
	Virus infectivity studies using HEK293T cells expressing ACE2 from 11 species of animals	Cat ACE2 could mediate SARS-CoV-2 entry	Tang et al., 2020
Swine (pigs)	Phylogenetic clustering and sequence alignment to evaluate the receptor-utilizing capability of ACE2	Swine ACE2 have the receptor-utilizing capability of SARS-CoV-2	Qiu et al., 2020
	Use expressed RBD proteins to perform surface staining of cells transfected with expression plasmids of ACE2 orthologs	Pigs support the efficient entry of SARS-CoV-2, SARS-CoV, and Bat-nCoV RaTG13	Li Y. J. et al., 2020
	Use single-cell technique to screen of ACE2 and TMPRSS2 (SARS-CoV-2 target cell) in different organs of animals	Pig have a variety of cell types co-expressing SARS-ACE2 and TMPRSS2	Chen D. S. et al., 2020
	Virus infectivity studies using HEK293T cells expressing ACE2 from 11 species of animals	Pig ACE2 could mediate SARS-CoV-2 entry	Tang et al., 2020
	Intranasally inoculated SARS-CoV-2 to domestic animals	**Oppose:** Pigs have little susceptibility to SARS-CoV-2	Shi et al., 2020
	Investigate the level of ACE2 expression in different organs	**Oppose:** Higher mRNA levels in organs such as kidney and heart, while low mRNA levels in respiratory tract	Zhai et al., 2020

## Author Contributions

B-pT contributed the conception and design of this review. JZ wrote the manuscript. B-pT and WC revised and edited this manuscript. All authors reviewed the draft and approved the submission.

## Conflict of Interest

The authors declare that the research was conducted in the absence of any commercial or financial relationships that could be construed as a potential conflict of interest.
