# Supplementary material for: The Potential Intermediate Hosts for SARS-CoV-2
Source: Front Microbiol. 2020 Sep 30;11:580137. doi: 10.3389/fmicb.2020.580137 (PMC7554366; doi:10.3389/fmicb.2020.580137)
Supplement: Supplementary file 1 [file Table_1.docx]

**Supplemental Table 1: The potential natural reservoirs of SARS-CoV-2**

| **Species** | **Analytic method** | **Supported evidence** | **Reference** |
| --- | --- | --- | --- |
| **Bat** | Sequence homology comparison at the whole-genome level | Bat-nCoV RaTG13 shared 96.2% sequence identity with SARS-CoV-2 | Zhou et al.  Nature 2020[1] |
|  | AI-enabled clustering algorithms analyze the raw sequences of SARS-CoV-2 | All examined SARS-CoV-2 virus genomes and bat-nCoV belongs to the same cluster with the closest relationship | Nguyen et al. bioRxiv 2020[2] |
|  | Compare the infectivity patterns by deep learning algorithm of VHP | Bat-nCoV have the closest infectious patterns to SARS-CoV-2 | Guo et al. bioRxiv 2020[3] |
|  | Phylogenetic tree analysis and structural models’  comparison. | The bat-nCoV isolates are confirmed to have close relationship with SARS-CoV-2 | Dabravolski et al. J Med Virol 2020 [4] |
| **Pangolin** | Phylogenetic analysis among Pangolin-nCoV, RaTG13 and SARS-CoV-2 | The five amino acid of RBD in Pangolin-nCoV is completely consistent to SARS-CoV-2 | Zhang et al. Current biology 2020 [5] |
|  | High-throughput  Sequencing and phylogenetic analysis | Pangolin-nCoV belongs to two sub-lineages of SARS-CoV-2 related coronaviruses | Lam et al.  Nature 2020 [6] |

**Reference**

1.Zhou P, Yang XL, Wang XG, Hu B, Zhang L, Zhang W, et al. A pneumonia outbreak associated with a new coronavirus of probable bat origin. *Nature.* 2020;579:270-273. doi:10.1038/s41586-020-2012-7

2.Nguyen TT, Abdelrazek M, Nguyen DT, Aryal S, Nguyen DT and Khatami A. Origin of Novel Coronavirus (COVID-19): A Computational Biology Study using Artificial Intelligence. *bioRxiv.* 2020:2020.05.12.091397. doi:10.1101/2020.05.12.091397

3.Guo Q, Li M, Wang C, Wang PH, Fang ZC, tan J, et al. Host and infectivity prediction of Wuhan 2019 novel coronavirus using deep learning algorithm. *bioRxiv.* 2020:2020.01.21.914044. doi:10.1101/2020.01.21.914044

4.Dabravolski SA and Kavalionak YK. SARS-CoV-2: Structural diversity, phylogeny, and potential animal host identification of spike glycoprotein. *J Med Virol.* 2020doi:10.1002/jmv.25976

5.Zhang T, Wu Q and Zhang Z. Probable Pangolin Origin of SARS-CoV-2 Associated with the COVID-19 Outbreak. *Current biology : CB.* 2020;30:1346-1351.e2. doi:10.1016/j.cub.2020.03.022

6.Lam TT, Shum MH, Zhu HC, Tong YG, Ni XB, Liao YS, et al. Identifying SARS-CoV-2 related coronaviruses in Malayan pangolins. *Nature.* 2020doi:10.1038/s41586-020-2169-0
